# Risk factors for developing hyperoxaluria in children with Crohn’s disease

**DOI:** 10.1007/s00467-022-05674-3

**Published:** 2022-07-08

**Authors:** Amr Salem, Happy Sawires, Ayman Eskander, Radwa Marwan, Engy Boshra

**Affiliations:** 1 Pediatric Nephrology Department, Cairo University, Cairo, Egypt; 2 Pediatric Gastroenterology Department, Cairo University, Cairo, Egypt; 3 Clinical and Chemical Pathology Department, Cairo University, Cairo, Egypt

**Keywords:** Crohn’s disease, Hyperoxaluria, Nephrolithiasis, Malabsorption, Urine oxalate/creatinine ratio

## Abstract

**Background:**

For the purpose of a better understanding of enteric hyperoxaluria in Crohn’s disease (CD) in children and adolescents, we investigated the occurrence and risk factors for development of hyperoxaluria in those patients.

**Methods:**

Forty-five children with CD and another 45 controls were involved in this cross-sectional study. Urine samples were collected for measurement of spot urine calcium/creatinine (Ur Ca/Cr), oxalate/creatinine (Ur Ox/Cr), and citrate/creatinine (Ur Citr/Cr) ratios. Fecal samples were also collected to detect the oxalyl-CoA decarboxylase of *Oxalobacter formigenes* by PCR. Patients were classified into 2 groups: group A (with hyperoxaluria) and group B (with normal urine oxalate excretion). The disease extent was assessed, and the activity index was calculated.

**Results:**

According to the activity index, 30 patients (66.7%) had mild disease and 13 patients (28.9%) had moderate disease. There was no significant difference in Ur Ox/Cr ratio regarding the disease activity index. *O. formigenes* was not detected in 91% of patients in group A while it was detected in all patients in group B (*p* < 0.001). By using logistic regression analysis, the overall model was statistically significant when compared to the null model, (*χ*^2^ (7) = 52.19, *p* < 0.001), steatorrhea (*p* = 0.004), frequent stools (*p* = 0.009), and *O. formigenes* (*p* < 0.001).

**Conclusion:**

Lack of intestinal colonization with *O. formigenes*, steatorrhea, and frequent stools are the main risk factors for development of enteric hyperoxaluria in CD patients. Identifying risk factors facilitates proper disease management in future studies.

**Graphical abstract:**

A higher resolution version of the Graphical abstract is available as Supplementary information

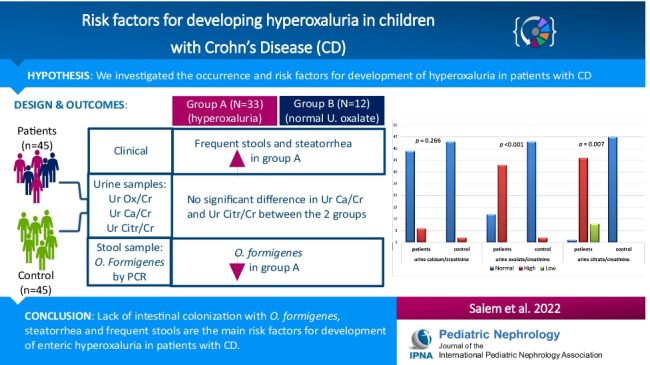

**Supplementary Information:**

The online version contains supplementary material available at 10.1007/s00467-022-05674-3.

## Introduction

Extraintestinal manifestations of Crohn’s disease (CD) can be divided into direct consequences (due to anatomical or metabolic complications caused directly by the disease itself) and reactive manifestations (associated with the inflammatory activity of the disease) [1]. Secondary or enteric hyperoxaluria is one of the frequent direct complications of CD and is well known to cause urolithiasis and nephrocalcinosis that less frequently may contribute to kidney failure [2].

The prevalence of enteric hyperoxaluria in children and adolescents with CD has not been precisely determined. However, it was reported that 52.2% of children and adolescents with CD had abnormal urinary oxalate excretion [3], and the prevalence of urolithiasis among adults with inflammatory bowel disease (IBD) is higher (ranging from 12 to 28%) than in the general population [4].

Enteric hyperoxaluria in patients with CD is multifactorial as it happens in patients with and without malabsorption. It is assumed to be secondary to high-solubility levels of oxalate inside the intestinal lumen, and the associated increase in bowel permeability to oxalate that results from excess bile salts and concomitant inflammation of colonic mucosa [5]. Prolonged steatorrhea associated with fat malabsorption may lead to low urine volume and consequent low urinary citrate which further increases the risk for stone formation [6]. Oxalate homeostasis could be affected by the gut microbiota and prolonged use of antibiotics. Certain bacterial strains, including *Lactobacillus* spp., *Oxalobacter* spp., and *Bifidobacterium* spp., can degrade oxalate and may be capable of modulating bowel permeability to oxalate [7, 8].

For the purpose of a better understanding of enteric hyperoxaluria in CD patients and ensuing better treatment, we investigated the occurrence and risk factors for development of hyperoxaluria in children and adolescents with CD. We also identified the association between different clinical and biochemical variables and the development of hyperoxaluria in those children.

## Methodology

In this cross-sectional analytical study, forty-five children and adolescents with CD (mean age 8.8 years, min 2.5 and max 15) were involved. The patients were recruited from the gastroenterology clinic at Cairo University Children’s Hospital. CD was diagnosed on the basis of both upper and lower gastrointestinal endoscopy and histopathology. The patients were eligible to participate in the study if CD diagnosis was fully established based on the above criteria and if at least 6 months had lapsed since the time of diagnosis. Patients who were offspring of consanguineous marriage; or with anuria, kidney failure, primary hyperoxaluria; or had history of urolithiasis prior to the onset of CD were excluded from the study. Another forty-five gender- and age-matched children were involved as a control group. Both patients and control groups were convenience samples. The control group was recruited from the pediatric surgery unit in the same hospital 2 days before doing minor surgery. None of the patients or controls had received antibiotics, vitamin C, or medications affecting lithogenicity of urine 4 weeks before inclusion. An informed written consent was obtained from patients’ caregivers. Our study was conducted in accordance with the Declaration of Helsinki and approved by a local ethical committee.

The following data were collected from the patients: age, gender, weight, height, family history of IBD, disease duration, activity, frequency of stooling, presence or absence of bleeding per rectum, abdominal pain, persistent diarrhea, and steatorrhea. Persistent diarrhea was defined as diarrhea more than 3 months. Patients were considered to have faltering growth if they fell across two or more weight centile spaces, and birth weight was between the 9th and 91st centiles [9]. Disease activity index was calculated according to The Pediatric Crohn Disease Activity Index (PCDAI) [10]. The extent of the disease was classified according to the Pediatric Modification of the Montreal Classification for Inflammatory Bowel Disease (PMMCIBD) [11].

All of the patients and the control group were instructed 1 month prior to the study about the dietary regimen to follow. The aim of such dietary regimen was to ensure oxalate intake was equal in both patients and the control group. Both groups were instructed by a dietician about the daily portions of the following foods (based on oxalate content/100 g [12]) not to be exceeded: spinach, chocolate, nuts, okra, tomatoes, French fries, tea, and legumes.

Blood samples were taken from the patients to measure complete blood count (CBC), erythrocyte sedimentation rate (ESR), C-reactive protein (CRP), serum sodium (Na), potassium (K), urea nitrogen (BUN), creatinine, uric acid, calcium (Ca), phosphorus (P), alkaline phosphatase (ALP), total bilirubin, albumin, and liver enzymes (AST, ALT, and GGT).

The second non-fasting mid-stream morning urine sample was collected from all of the children for measurement of spot urine calcium/creatinine (Ur Ca/Cr), oxalate/creatinine (Ur Ox/Cr), and citrate/creatinine (Ur Citr/Cr) ratios. Spot urine samples were collected in containers without any additional preservatives. All the ratios were presented as mg/mg. Normal values for spot urine samples were assessed on the basis of the following references [13–15].

Abdominal ultrasound examination was carried out on the same day by an expert radiologist for detection of urolithiasis or nephrocalcinosis.

Ur Ca/Cr ratio was obtained via a Beckman Coulter AU680 (Beckman Coulter, Kraemer Blvd Brea, CA 92,821, USA) while Ur Ox/Cr and Ur Citr/Cr ratios were obtained via a Riele 5010 photometer semi-automated clinical chemistry analyzer (Robert Riele GmbH & Co KG, Kurfuerstenstrasse, Berlin, Germany).

For confirmation, 24-h urine was collected from patients for measuring oxalate. After collecting urine in containers containing 20 ml of HCl as preservative (final pH was between 1.5 and 2.0), the aliquots were stored at − 20 °C until analysis. The 24-h urinary oxalate was done using a Quantitative UV Oxalate Assay (BEN — Biochemical Entreprise Milano — Italy). This was carried out on a Riele 5010 photometer semi-automated clinical chemistry analyzer (Robert Riele GmbH & Co KG, Kurfuerstenstrasse, Berlin, Germany).

Patients were classified into two groups: group A (with hyperoxaluria) and group B (with normal urine oxalate excretion). Patients with hyperoxaluria were asked for two further spot urine samples on two different days for assessment of Ur Ox/Cr ratio for confirmation.

Fecal samples were collected from patients and the control group and stored at − 80 °C until DNA extraction using QIAamp Fast DNA stool for pathogen detection (*Oxalobacter formigenes*). To detect the oxalyl-CoA decarboxylase of *O. formigenes*, polymerase chain reaction (PCR) was performed using QuantiNova SYBR Green PCR.

The primary outcome was to identify the predictors associated with hyperoxaluria in children and adolescents.

### Statistical analysis

Quantitative data were presented as mean ± SD (standard deviation) if parametric and median and interquartile range values skewed from normal variation. Qualitative data were presented as frequencies and percentages. Independent *t* test and the Mann–Whitney test were used to compare parametric and non-parametric variables, respectively. The difference between categorical variables was analyzed using the chi-square test. The Pearson correlation coefficients were used to determine significant correlations between quantitative data. Binary logistic regression analysis was used to detect predictors of hyperoxaluria. The significance level was set at *p* < 0.05. Statistical analysis was performed with SPSS 26.0 (Statistical Package for Scientific Studies) for Macintosh.

## Results

**Fig. 1:**
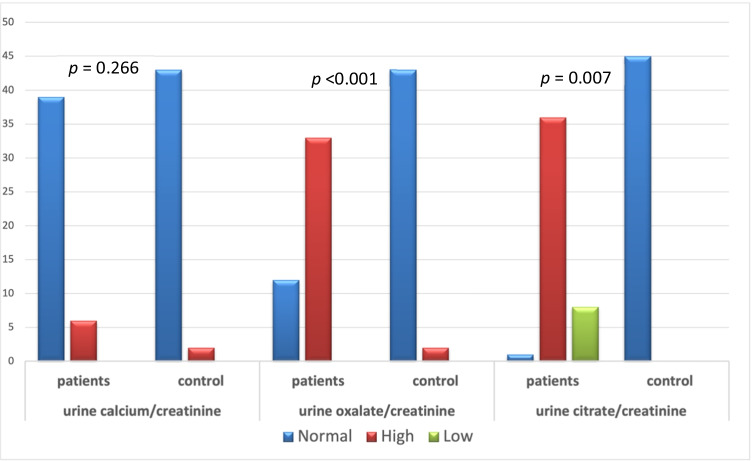
Urinary calcium, oxalate, and citrate excretion in patients and control group (percentage)

We enrolled 45 patients with CD; among them, 33 patients (73.3%) had hyperoxaluria (group A) and 12 patients (26.7%) had normal urine oxalate excretion (group B). We also enrolled 45 gender- and age-matched controls, none of whom had hyperoxaluria. Only 5 patients (11.1%) had kidney stones detected by abdominal ultrasonography. The demographic data of patient and control groups are shown in Table 1 and Fig. 1.

**Table 1:** Demographic data of the study population

	Patients (*N* = 45)	Control (*N* = 45)	*p* value
Age (years)	8.86 ± 4.05 (min 2.5, max 15)	6.91 ± 3.86 (min 3.1, max 15)	0.149
Gender			
Male	26 (58%)	25 (56%)	0.937
Female	19 (42%)	20 (44%)	
Weight (z score)	− 0.88 ± 1.31 (min − 3.95, max 1.58)	− 0.08 ± 1.47 (min − 1.66, max 1.93)	< 0.001
Height (z score)	− 1.02 ± 1.35 (min − 4.94, max 2.01)	− 0.26 ± 1.1 (min − 1.38, max 1.86)	< 0.001
Family history of IBD			
Yes	8 (17.8%)		
No	37 (82.2%)		
Disease duration (years)	2.54 ± 1.72 (min 0.1, max 7)		
Persistent diarrhea			
Yes	23 (51.1%)		
No	22 (48.9%)		
Frequent stools (> 5/day)			
Yes	15 (33.3%)		
No	30 (66.7%)		
Steatorrhea			
Yes	4 (8.9%)		
No	41 (91.1%)		
Faltering growth			
Yes	28 (62.2%)		
No	17 (37.8%)		
Abdominal pain			
Yes	44 (97.8%)		
No	1 (2.2%)		
Bleeding/rectum			
Yes	36 (80%)		
No	9 (20%)		
Activity index			
Inactive	1 (2.2%)		
Mild	30 (66.7%)		
Moderate	13 (28.9%)		
Severe	1 (2.2%)		
Ur Ca/Cr (mg/mg)			
Normal	39 (86.7%)	43 (95.6%)	0.266
High	6 (13.3%)	2 (4.4%)	
Ur Ox/Cr (mg/mg)			
Normal	12 (26.7%)	45 (100%)	< 0.001
High	33 (73.3%)	0 (0%)	
Ur Citr/Cr (mg/mg)			
Normal	36 (80%)	45 (100%)	0.007
High	1 (2.2%)	0	
Low	8 (17.8%)	0	
*O. formigenes*			
Detected	15 (33.3%)	41 (91.1%)	< 0.001
Not-detected	30 (66.7%)	4 (8.9%)

According to PMMCIBD, 34 patients (75.6%) had A1a while 11 patients (24.4%) had A1b. All patients had ileocolonic (L3) CD. Regarding behavior, 41 patients (91.1%) were non-stricturing while only 4 patients (8.9%) were stricturing. A comparison of the 2 groups of patients is shown in Table 2.

**Table 2:** Comparison of patients with hyperoxaluria and patients with normal urine oxalate excretion

	Group A (*N* = 33)	Group B (*N* = 12)	*p* value
Age (years)	9.13 ± 4.25	6.91 ± 3.86	0.621
Gender			
Male	18 (54.5%)	4 (33.3%)	0.517
Female	15 (45.8%)	8 (66.7%)	
Weight (z score)	− 1.09 ± 1.32 (min.-3.95, max.1.58)	− 0.33 ± 1.14 (min − 2.27, max 1.40)	0.100
Height (z score)	− 1.11 ± 1.43 (min − 4.94, max 0.94)	− 0.78 ± 1.13 (min − 2.30, max 2.01)	0.663
Family history of IBD			
Yes	6 (18.2%)	2 (16.7%)	1
No	27 (81.8%)	10 (83.3%)	
Disease duration (years)	2.48 ± 1.84	2.36 ± 1.42	0.990
Persistent diarrhea			
Yes	19 (57.6%)	4 (33.3%)	0.189
No	14 (42.4%)	8 (66.7%)	
Frequent stools (> 5/day)			
Yes	17 (51.5%)	1 (8.3%)	0.014
No	16 (48.5%)	11 (91.7%)	
Steatorrhea			
Yes	15 (48.4%)	0 (0%)	< 0.001
No	18 (51.6%)	12 (100%)	
Faltering growth			
Yes	22(66.7%)	6 (50%)	0.325
No	11 (33.3%)	6 (50%)	
Abdominal pain			
Yes	33 (100%)	11 (91.7%)	0.267
No	0 (0%)	1 (8.3%)	
Bleeding/rectum			
Yes	25 (75.8%)	11 (91.7%)	0.407
No	8 (24.2%)	1 (8.3%)	
Activity index			
Inactive	0 (0%)	1 (8.3%)	
Mild	22 (66.7%)	8 (66.7%)	0.362
Moderate	10 (30.3%)	3 (25%)	
Severe	1 (3%)	0 (2.2%)	
Treatment modality			
Mesalazine + AZA	24 (72%)	9 (75%)	0.936
Mesalazine + AZA + steroids	4 (12%)	1 (8.3%)	
Biological treatment	5 (16%)	2 (16.7%)	
Kidney stones			
Yes	5 (15.2%)	0 (0%)	0.303
No	28 (84.8%)	12 (100%)	
Urine oxalate excretion (mmol/1.73 m^2^/day)	0.639 ± 0.1	0.216 ± 0.05	< 0.001
Ur Ca/Cr (mg/mg)			
Normal	4 (12.1%)	2 (16.7%)	0.650
High	29 (87.9%)	10 (83.3%)	
Ur Citr/Cr (mg/mg)			
Normal	28 (84.8%)	8 (66.7%)	0.309
High	0 (0%)	1 (8.3%)	
Low	5 (15.2%)	3 (25%)	
*O. formigenes*			
Detected	3 (9%)	12 (100%)	< 0.001
Not-detected	30 (91%)	0 (0%)	
Hemoglobin (gm/dl)	10.98 ± 1.71	11.16 ± 1.53	0.598
Platelets (× 1000/mm^3^)	310.76 ± 118.59	310.0 ± 89.86	0.939
TLC (/mm^3^)	8.10 ± 4.46	8.07 ± 4.34	0.878
ESR (/mm^3^)	21.82 ± 21.01	20.17 ± 13.70	0.797
CRP (mg/dl)	8.75 ± 18.69	4.19 ± 5.23	0.817
Na (meq/L)	138.55 ± 2.71	138.42 ± 3.03	1
K (meq/L)	4.11 ± 0.28	4.28 ± 0.33	0.181
BUN (mg/dl)	12.45 ± 5.46	12.33 ± 3.97	0.606
Creatinine (mg/dl)	0.55 ± 0.16	0.54 ± 0.15	0.666
Albumin (gm/dl)	4.09 ± 0.48	4.20 ± 0.27	0.279
Total bilirubin (mg/dl)	1.92 ± 0.19	0.38 ± 0.16	0.034
AST (mg/dl)	30.55 ± 18.90	24.92 ± 8.84	0.269
ALT (mg/dl)	21.0 ± 13.38	17.17 ± 7.0	0.410
GGT (mg/dl)	23.76 ± 60.22	12.25 ± 2.96	0.660
Calcium total (mg/dl)	8.98 ± 0.92	9.63 ± 0.68	0.028
Phosphorus (mg/dl)	4.74 ± 0.89	4.67 ± 0.62	0.887
ALP (IU/L)	229.12 ± 42.30	198.83 ± 29.86	0.008

**Fig. 2:**
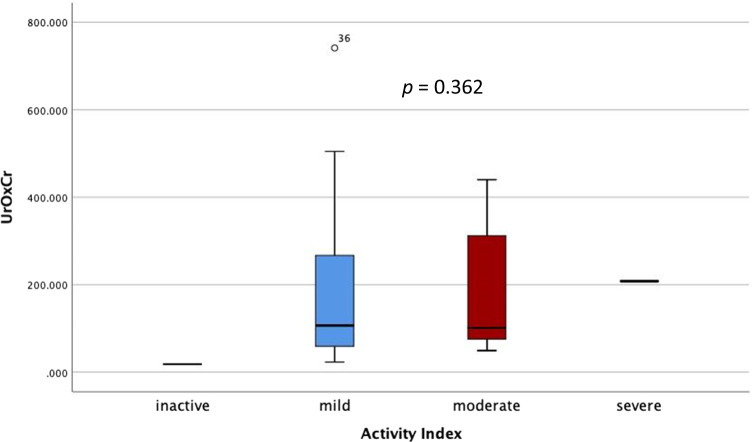
Boxplot of means of urine oxalate/creatinine ratio according to activity index in patient group

According to the activity index, 30 patients (66.7%) had mild disease and 13 patients (28.9%) had moderate disease. We did not observe any significant difference in the Ur Ox/Cr ratio regarding disease activity index (*p* = 0.341) (Fig. 2).

The majority of the patients were on mesalazine and azathioprine (72% in group A and 75% in group B). We did not observe any significant difference between the 2 groups regarding treatment modality (*p* = 0.936).

We reported only 5 CD patients (11.1%) with kidney stones (all of them in group A), but we did not report any patient with nephrocalcinosis. The fact that all patients with kidney stones had hyperoxaluria and the non-significant difference between the 2 groups (*p* = 0.303) were attributed to the small number of cases of kidney stones. There was a significant difference between patients with and without kidney stones regarding the Ur Citr/Cr ratio (*p* < 0.001) and steatorrhea (*p* = 0.036) with lower Ur Citr/Cr ratios in patients with kidney stones (41.80 ± 34.00).

*O. formigenes* was not detected in 91% of patients with hyperoxaluria (group A) while it was detected in all patients in group B (*p* < 0.001). On the other hand, there was no significant difference between patients with and without kidney stones regarding detection of *O. formigenes* (*p* = 0.651).

**Fig. 3:**
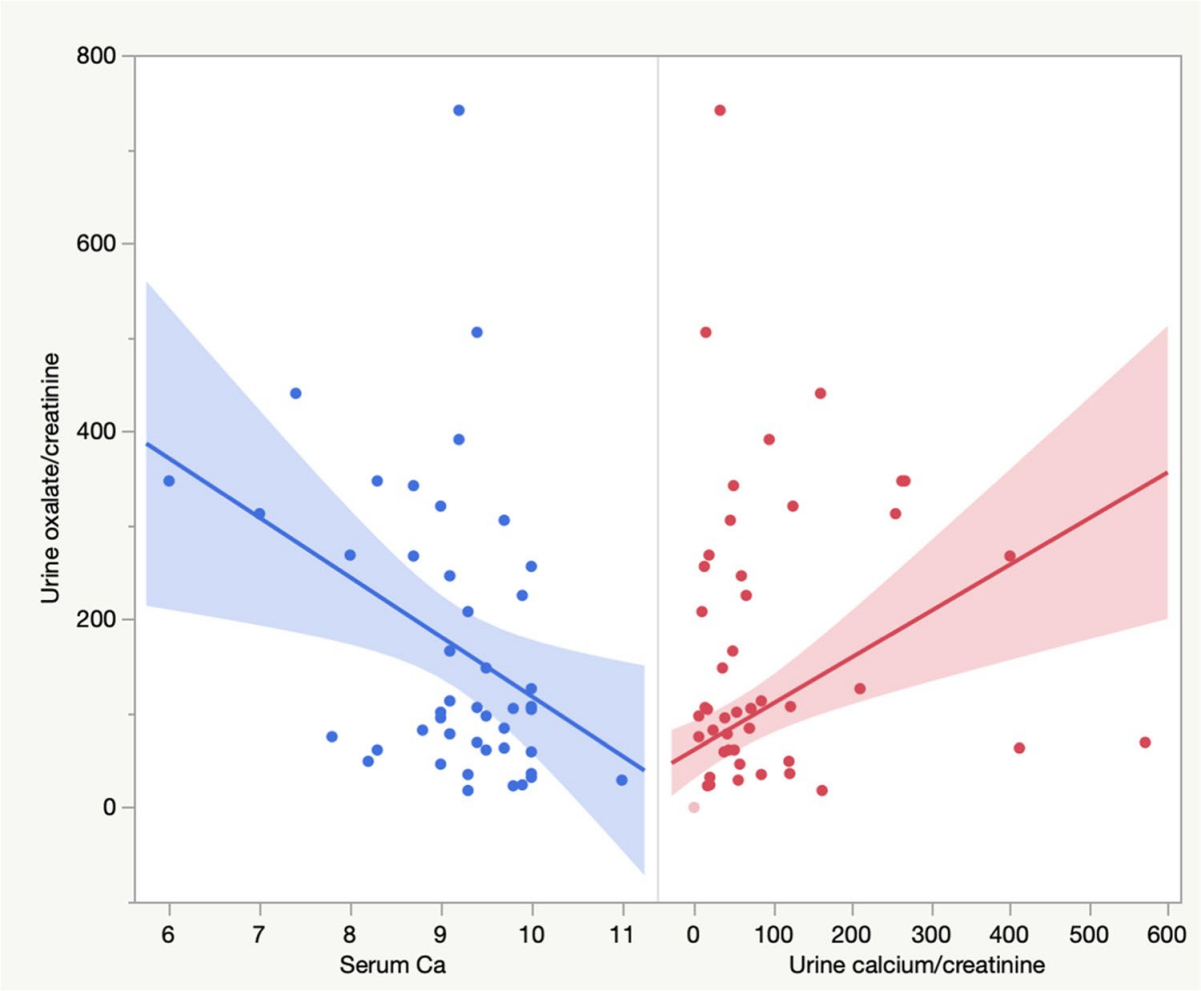
Correlations between serum calcium and urine calcium/creatinine ratio with urine oxalate/creatinine ratio

There was a negative significant correlation between serum Ca and Ur Ox/Cr ratio (*p* = 0.013) and positive significant correlation between Ur Ox/Cr and Ur Ca/Cr ratios (*p* = 0.001) (Fig. 3). Regarding the other clinical and biochemical variables, there were no significant correlations with Ur Ox/Cr (*p* > 0.05).

A logistic regression was carried out to assess the effect of persistent diarrhea, frequency of stools, steatorrhea, activity index, and detection of *O. formigenes* on the likelihood of hyperoxaluria. The overall model was statistically significant when compared to the null model, (χ^2^ (7) = 52.19, *p* < 0.001), explained 100% of the variation of hyperoxaluria (Nagelkerke R2), and correctly predicted 73.3% of cases. Steatorrhea (*p* = 0.004), frequent stools (*p* = 0.009), and *O. formigenes* (*p* < 0.001) were significant, but persistent diarrhea (*p* = 0.150) and activity index (*p* = 0.094) were not. The odds of hyperoxaluria were 8.

## Discussion

Enteric hyperoxaluria is a complex multifactorial disease with significant consequences for patient quality of life [2]. In the present study, we found a relatively higher incidence (73%) of enteric hyperoxaluria in children and adolescents with CD compared to a single previous study done with pediatric patients (52.2%) [3]. In another study, Mc Connell et al. found that 36% of adult CD patients had hyperoxaluria, and they detected kidney calculi in only 2 out of 25 patients [16]. The variation in incidence is mostly due to the population mix in the different studies.

Not all patients with enteric hyperoxaluria in this study developed nephrolithiasis. Only 11% of the study population (5 out of 33 with hyperoxaluria) had nephrolithiasis which is higher than its incidence in a previous study of pediatric patients (0.37–1%) [17] but comparable with adult patients with IBD (9–18%) [18, 19]. Multiple factors contribute to the development of nephrolithiasis in CD patients. Urine volume, pH and urinary calcium, oxalate, magnesium, and citrate excretion are identified as perpetrators in stone formation, but the contribution of each factor is still debatable [20]. We studied only a few parameters and found that the main contributor for development of nephrolithiasis among hyperoxaluria patients in CD was low urinary citrate. Though it is not always adequately recognized as a risk factor, citrate is the main inhibitor of calcium and oxalate crystallization, and it is commonly seen to be low in patients with gastrointestinal malabsorption [21].

These findings question the importance related to hyperoxaluria alone in predisposing patients with CD to nephrolithiasis. Hyperoxaluria by itself does not inevitably give rise to stones, and other factors may contribute.

Recognizing risk factors and understanding the impact of CD on the development of enteric hyperoxaluria is an important aspect of disease management. We identified three main risk factors for development of enteric hyperoxaluria in children and adolescents with CD: steatorrhea, frequent stools, and lack of intestinal colonization by *O. formigenes.*

In patients with CD, as part of fat malabsorption, increased fatty acids might compete with oxalate for calcium binding which leads to increased soluble oxalate that is ready for absorption with an increased risk of hyperoxaluria [22]. The present study confirmed the association between steatorrhea and frequent stools, as pathogenetic mechanisms, and development of hyperoxaluria in children with CD.

In addition to the aforementioned pathogenetic mechanism, we found a significant proportion of CD patients with less *O. formigenes* colonization compared with controls, and the majority of the hyperoxaluria group (91%) had undetected *O. formigenes* in their stools. This is in accordance with a recent 2021 study analyzing large-scale data demonstrating that in comparison with the general population, patients with oxalate stones have low levels of intestinal colonization with *O. formigenes* [23].

Oxalates are the primary source of energy for *Oxalobacter formigenes* capable of degrading them, and they are believed to be a line of defense against stone formation [24]. From the present study, we could not determine the exact reason for low intestinal colonization with *O. formigenes* in CD patients. However, this could be attributed to the combination of intestinal microbiota with intestinal inflammation in CD patients and breakage of local tolerance to intestinal flora [25].

In the present study, we did not find any significant link between CD activity or disease duration and development of enteric hyperoxaluria. This is in discordance with a retrospective cohort study published in 2017 which found that IBD activity was a significant risk factor for the development of hyperoxaluria and kidney stones in the study population [19]. Disease activity was considered as a risk factor for development of kidney stones in ulcerative colitis and not CD, and this was confirmed by another study of 168 patients (93 CD and 75 ulcerative colitis) [18].

There was no significant link between treatment modality and development of enteric hyperoxaluria in the present study. Crystallization of sulfasalazine metabolites which occurs within the urinary collecting system may predispose to nephrolithiasis in CD patients, but it was not reported to predispose to hyperoxaluria [26].

Urinary calcium excretion was not significantly higher in Crohn’s disease patients compared to the control group, and this confirms several previous studies [20, 27, 28]. Interestingly, however, we observed higher urinary calcium excretion in patients with enteric hyperoxaluria, and this finding needs to be studied further. Being a granulomatous disorder, hypercalcemia is theoretically suspected in CD patients as a consequence of altered vitamin D metabolism, but hypocalcemia is seen more commonly in those patients [29]. The negative correlation between serum calcium and Ur Ox/Cr ratio in this study could be explained by the lack of calcium and vitamin D supplementation prior to the study in addition to decreased intestinal calcium absorption and steroid therapy which interferes with intestinal calcium transport. Also, urinary citrate excretion was not significantly lower in CD patients compared to the control group. In another study of 86 adult patients with CD, urinary citrate was significantly lower compared to healthy controls [30]. Tubular reabsorption of citrate is higher in patients with malabsorption syndromes compared to those without malabsorption syndromes [31].

To the best of our knowledge, this is the first study to identify risk factors for development of hyperoxaluria in children with CD. However, our results should be interpreted in the context of some limitations. First, reliance on random Ur Ox/Cr, Ur Ca/Cr, and Ur Citr/Cr ratios rather than 24-h urine collection for our results, though let us admit the difficulty of 24-h urine collection in children. Second, limiting the diet of both patient and control groups to the lower normal range of dietary oxalate intake may not reflect their normal diet and may mask hyperoxaluria in some cases. Third, we could not eliminate the effect of medications used to control disease activity. Furthermore, 62% of the patients had faltering growth. These patients may have had reduced muscle mass with low creatinine which may be misleading in spot urine sample results.

Identifying risk factors smooths the path for proper disease management. For example, introduction of oxalate degradation through colonization with *O. formigenes* should have a distinct effect on lowering absorption of oxalate.

In conclusion, lack of intestinal colonization with *O. formigenes*, steatorrhea, and frequent stools are the main risk factors for development of enteric hyperoxaluria in children and adolescents with CD. Identifying risk factors smooths the way for proper disease management in future studies.

## Supplementary Information

Graphical Abstract (PPTX 296 KB)
