# Supplementary material for: Risk factors for developing hyperoxaluria in children with Crohn’s disease
Source: Pediatr Nephrol. 2022 Jul 8;38(3):781–9. doi: 10.1007/s00467-022-05674-3 (PMC9842562; doi:10.1007/s00467-022-05674-3)
Supplement: Supplementary file 1 — (PPTX 296 KB) [file 467_2022_5674_MOESM1_ESM.pptx]

## Slide 1
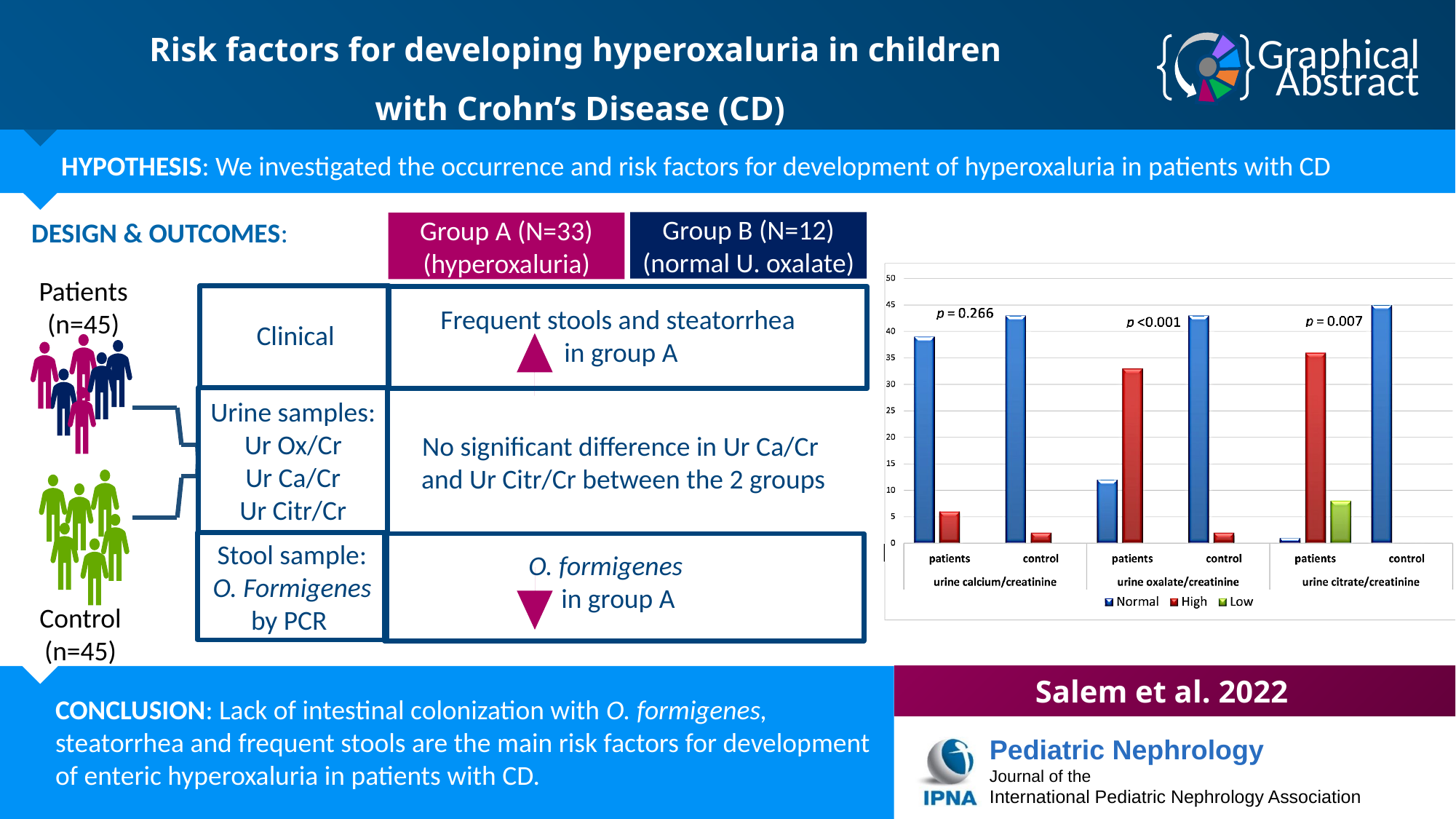

Risk factors for developing hyperoxaluria in children
with Crohn’s Disease (CD)
HYPOTHESIS: We investigated the occurrence and risk factors for development of hyperoxaluria in patients with CD
DESIGN & OUTCOMES:
Group B (N=12)
(normal U. oxalate)
Group A (N=33)
(hyperoxaluria)
Patients (n=45)
Frequent stools and steatorrhea
in group A
Clinical
Urine samples:
Ur Ox/Cr
Ur Ca/Cr
Ur Citr/Cr
No significant difference in Ur Ca/Cr
and Ur Citr/Cr between the 2 groups
Stool sample:
O. Formigenes by PCR
O. formigenes
 in group A
Control (n=45)
Salem et al. 2022
CONCLUSION: Lack of intestinal colonization with O. formigenes, steatorrhea and frequent stools are the main risk factors for development of enteric hyperoxaluria in patients with CD.
